# Superior metal artifact reduction of tin-filtered low-dose CT in imaging of lumbar spinal instrumentation compared to conventional computed tomography

**DOI:** 10.1007/s00256-023-04467-5

**Published:** 2023-10-07

**Authors:** Christoph Stern, Florian Wanivenhaus, Andrea B. Rosskopf, Mazda Farshad, Reto Sutter

**Affiliations:** 1 Radiology, Balgrist University Hospital, Forchstrasse 340, 8008 Zurich, Switzerland; 2 Faculty of Medicine, University of Zurich, Zurich, Switzerland; 3 Department of Orthopaedic Surgery, Balgrist University Hospital, Forchstrasse 340, 8008 Zurich, Switzerland

**Keywords:** Tomography, X-ray computed, Spine, Pedicle screws, Tin, Radiation dosage

## Abstract

**Objective:**

To compare the image quality of low-dose CT (LD-CT) with tin filtration of the lumbar spine after metal implants to standard clinical CT, and to evaluate the potential for metal artifact and dose reduction.

**Materials and methods:**

CT protocols were optimized in a cadaver torso. Seventy-four prospectively included patients with metallic lumbar implants were scanned with both standard CT (120 kV) and tin-filtered LD-CT (Sn140kV). CT dose parameters and qualitative measures (1 = worst,4 = best) were compared. Quantitative measures included noise, signal-to-noise ratio (SNR), contrast-to-noise ratio (CNR), and the width and attenuation of the most prominent hypodense metal artifact. Standard CT and LD-CT were assessed for imaging findings.

**Results:**

Tin-filtered LD-CT was performed with 60% dose saving compared to standard CT (median effective dose 3.22 mSv (quartile 1–3: 2.73–3.49 mSv) versus 8.02 mSv (6.42–9.27 mSv; *p* < .001). Image quality of CT and tin-filtered low-dose CT was good with excellent depiction of anatomy, while image noise was lower for CT and artifacts were weaker for tin-filtered LD-CT. Quantitative measures also revealed increased noise for tin-filtered low-dose CT (41.5HU), lower SNR (2) and CNR (0.6) compared to CT (32HU,3.55,1.03, respectively) (all *p* < .001). However, tin-filtered LD-CT performed superior regarding the width and attenuation of hypodense metal artifacts (2.9 mm and -767.5HU for LD-CT vs. 4.1 mm and -937HU for CT; all *p* < .001). No difference between methods was observed in detection of imaging findings.

**Conclusion:**

Tin-filtered LD-CT with 60% dose saving performs comparable to standard CT in detection of pathology and surgery related complications after lumbar spinal instrumentation, and shows superior metal artifact reduction.

**Supplementary Information:**

The online version contains supplementary material available at 10.1007/s00256-023-04467-5.

## Introduction

Image artifacts and a high radiation dose are major concerns for computed tomography (CT) evaluation of the lumbar spine after instrumentation surgery. Lumbar instrumentation is a very common surgical procedure in degenerative diseases of the lumbar spine and postoperative evaluation is routinely performed by standard radiographs as first-line imaging, usually with anteroposterior and lateral views [1, 2]. In a subset of patients, especially with persistent pain, an additional CT scan is performed to exclude fractures, misplacement or loosening of metal implants [3]. However, this results in a much higher total radiation dose for the patient compared to the evaluation with radiographs, despite the benefit of detailed cross-sectional anatomic information provided by the CT [4, 5].

Besides well-established techniques such as automatic tube voltage and current modulation and iterative image reconstruction, tin prefiltration is a fairly new advancement in CT imaging to further reduce the radiation dose. Tin prefiltration CT uses an additional tin filter after the X-ray tube to shape the photon spectrum of the X-ray beam. The tin filter hardens the X-ray spectrum by filtering out low energy photons, which contribute little to imaging of high contrast structures (e.g., bone, metal) due to absorption. The result is more penetrable photons and a reduced radiation dose for the patient [6, 7]. Tin prefiltration CT showed substantial radiation dose reduction without compromise in image quality for the chest [8–10] and abdomen [11]. Stern et. al showed the feasibility of tin-filtered low-dose CT (LD-CT) of the pelvis with a radiation dose equivalent to standard radiographs. Furthermore, the study discovered less image artifacts (scattering, beam hardening) for the tin-filtered LD-CT compared to the standard CT [10.1007/s00330-021-07824-x].

To our knowledge, the combination of tin prefiltration and low-dose CT imaging has not been evaluated in patients with metal implants of the lumbar spine for the ability to detect pathology and surgery related complications. Tin prefiltration CT without and with iterative metal artifact reduction was only evaluated in cadaver studies with metal implants [12, 13] and in 9 patients with metallic implants of the lumbar spine scanned on a photon-counting-detector CT but not on a conventional energy-integrating-detector CT [14]. We hypothesize that it is feasible to both reduce artifacts around metal implants of the lumbar spine and reduce the radiation dose when applying a low-dose CT protocol with tin filtration in clinical routine, while maintaining diagnostic accuracy.

Therefore, the purpose of the study was to compare the image quality of tin-filtered LD-CT of the lumbar spine with metal implants to standard clinical CT, and to evaluate the potential for metal artifact and dose reduction.

## Materials and methods

This prospective single-center study was approved by the cantonal ethics committee. The study was in accordance with the principles of Good Clinical Practice, the Declaration of Helsinki, and other Swiss regulations. For the cadaver, the permission for scientific use existed. All participating patients gave their written informed consent prior to inclusion.

### Instrumented cadaver

One intact torso with instrumented lumbar pedicle screws was used for optimization of CT parameters. With the experience from our previous study [10.1007/s00330-021-07824-x], a tin-filtered low-dose CT protocol for the instrumented spine was established, which was applied to all participants.

### Study participants

Patients (male and female) aged 18 years or older with metal implants of the lumbar spine and a clinically indicated CT examination of the lumbar spine at Balgrist University Hospital were prospectively included. Exclusion criteria were tumor or pregnancy. The study comprised the period January 2021 to September 2021.

### CT imaging technique

The clinically indicated non-contrast standard CT without tin filtration of the instrumented lumbar spine was performed on a 128-slice CT scanner (SOMATOM Edge Plus, Siemens Healthineers, Erlangen, Germany). For all standard CT scans automated tube current modulation (CARE Dose4D, reference 250 mAs) was activated, tube voltage was set to 120 kV and further parameters were a collimation width of 0.6 mm, a rotation time of 1 s and a pitch of 0.8.

Immediately following the standard CT, all study participants were additionally scanned over the identical coverage in z-axis with the non-contrast tin-filtered low-dose CT protocol on the same CT machine. Parameters of the tin-filtered LD-CT scan protocol were: fixed tube voltage (Sn 140 kV), active automated tube current modulation (CARE Dose4D, reference 250 mAs), a collimation width of 0.6 mm, a rotation time of 1 s and a pitch of 0.8.

### Image reconstruction

For both, the standard CT and the tin-filtered low-dose CT, image reconstruction in bone kernel (Br 57) was performed in the following planes: axial (2 mm), coronal (3 mm) and sagittal (3 mm). Furthermore, axial images with a 1-mm section thickness were reconstructed in soft tissue kernel (Br 38) for both datasets. For all image reconstructions advanced modeled iterative reconstruction (ADMIRE) strength level 3 was used. Reconstructed images in bone kernel (Br 57) of both the standard CT and the tin-filtered LD-CT were displayed with a window width of 2500 HU and a window level of 600 HU, while the window width and level of images in soft tissue kernel (Br 38) were set to 400 HU and 60 HU, respectively.

### CT image interpretation

Standard CT and tin-filtered low-dose CT images were anonymized and interpreted independently by a fellowship-trained musculoskeletal radiologist [C.S. (reader 1)] with 9 years of experience and a fellowship-trained orthopedic spine surgeon [F.W. (reader 2)] with 10 years of experience. Image display and interpretation was in random order on a PACS workstation. Readers were blinded to each other and were blinded to clinical information and imaging results.

### Qualitative image analysis

On a 4-point Likert scale, reader 1 and 2 rated independently the depiction of anatomy (1 = poor, 2 = fair, 3 = moderate, 4 = good), image noise (1 = very high, 2 = high, 3 = moderate, 4 = minimal) and image artifacts (1 = very strong, 2 = strong, 3 = moderate, 4 = weak) for standard CT and tin-filtered LD-CT, respectively. The exact definition of each category is listed in supplementary table 1.

### Quantitative image analysis

The volume CT dose index (CTDIvol), dose length product (DLP), tube voltage (kV) and tube current–time product (mAs) were available for every examination and were extracted from the dose report of the standard CT and tin-filtered LD-CT, respectively. For every examination the scan length was calculated: scan length = DLP / CTDIvol.

In order to get an estimate of the effective dose, the DLP and a standard conversion factor *k* for the adult lumbar spine (0.018 mSv/mGy*cm) were used: effective dose = DLP * *k* [15].

**Fig. 1:**
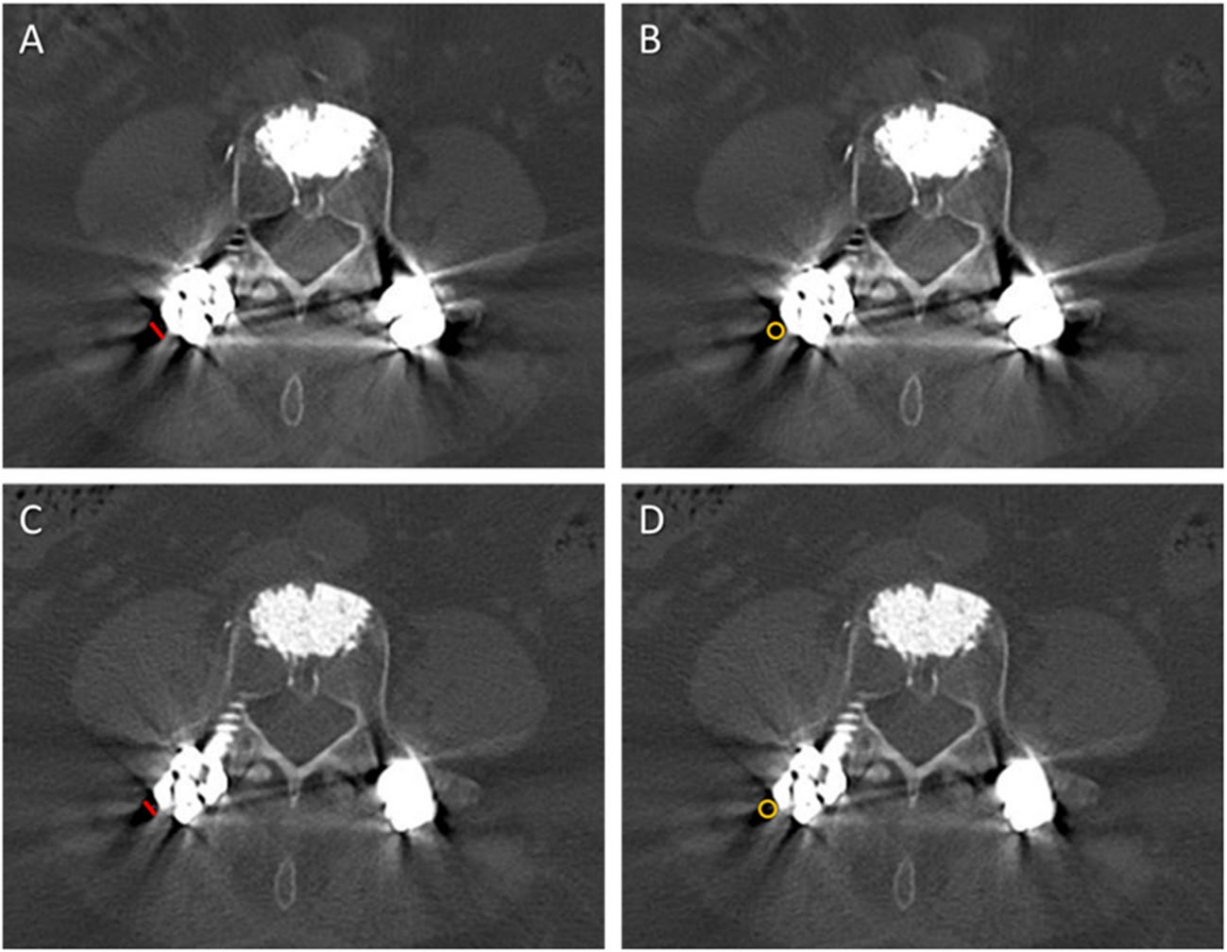
Measurements of the width and CT value of the most prominent hypodense artifact around the metallic spine implant, exemplary in a 74-year-old female with cement-augmented lumbar instrumentation. Reformatted axial CT image of the standard CT with 120 kV (**A** and **B**) and of the low-dose CT with tin filtration (Sn 140 kV; **C** and **D**) both demonstrate the measurement of the width (greatest diameter in millimeter; red line in **A** and **C**) and of the attenuation (region of interest; yellow circle in **B** and **D**) of the most prominent hypodense artifact around the metallic spine implant

For both the standard CT and the tin-filtered LD-CT, reader 1 measured the following CT values (HU) on axial images (2 mm) in bone kernel (Br 57) using region of interests (ROIs): trabecular bone (mean and standard deviation (SD); average of 2 measurements on 2 different slices at level L3), psoas muscle (mean; average of 2 measurements on the same slices as for trabecular bone) and subcutaneous fat (SD; average of 2 measurements on the same slices as for trabecular bone). All ROIs were placed in regions free of metal artifacts caused by spine implants. Furthermore, for both datasets the width of the most prominent hypodense artifact around the metallic spine implant was measured and a ROI was placed to record the attenuation. The sizes of the ROIs were chosen appropriately in order to measure the greatest possible dimension of each category (Fig. 1). Equally sized ROIs were used for the averaged measurement of trabecular bone, psoas muscle and subcutaneous fat, respectively.

Image noise was defined as the averaged standard deviation of the CT attenuation in subcutaneous fat [14]. For trabecular bone the signal-to-noise ratio (SNR), contrast-to-noise ratio (CNR) and a figure of merit (FOM; to compare the dose efficiency between protocols by normalizing the CNR) were calculated for both the standard CT and the tin-filtered LD-CT using the following equations:

SNR = (mean_trabecular bone / SD_subcutaneous fat); CNR = (mean_ trabecular bone – mean muscle) / (SD_trabecular bone); FOM = CNR^2^ / effective dose [11].

### Imaging findings

Standard CT and tin-filtered LD-CT were evaluated by both readers for the presence or absence of the following imaging findings: pedicle screw associated fracture, fracture at other location, fracture of pedicle screw, osteolytic bone resorption around pedicle screw, loosening of pedicle screw, segmental osseous fusion, pedicle screw traversing lateral recess.

### Statistical analysis

General descriptive statistics were applied. Ordinal data was reported as median with 25^th^ percentile (Q1) and 75^th^ percentile (Q3), and continuous data as mean with standard deviation (SD). To test for normal distribution the Shapiro–Wilk test was applied.

The Wilcoxon signed-rank test was used for comparison of CT dose parameters (scan length, CTDIvol, DLP, effective dose), quantitative parameters (CT values of trabecular bone, muscle, and hypodense artifact; noise, SNR, CNR and FOM) and qualitative parameters (depiction of anatomy, image noise, image artifacts) between standard CT and tin-filtered LD-CT.

Calculation of the prevalence of each imaging finding (pedicle screw associated fracture, fracture at other location, fracture of pedicle screw, osteolytic bone resorption around pedicle screw, loosening of pedicle screw, segmental osseous fusion, pedicle screw traversing lateral recess) was performed for standard CT and tin-filtered LD-CT, respectively, and the McNemar test was applied for comparison.

Agreement between readers was assessed with kappa statistics (ĸ*)* and effect size for ĸ was interpreted as slight (0–0.20), fair (0.21–0.40), moderate (0.41–0.60), substantial (0.61–0.80), or excellent (0.81–1.00) [16].

SPSS (Version 26, IBM Corporation, Armonk, NY) was used for statistical analysis. Significance was assumed for any value of *p* < 0.05.

## Results

### Study participants

Seventy-four patients with metal implants (42 males, 32 females, mean age 65.4 years ± SD 13.4 years) were prospectively included and all received standard CT and tin-filtered low-dose CT of the lumbar spine. The body mass index was available in 64 of 74 patients and was mean 26.7 ± SD 4.2. A total number of 419 vertebra (the sacrum was counted as 1 vertebra) and of 451 pedicle screws were evaluated.

### CT parameters and effective dose

With median 3.22 mSv (Q1–Q3: 2.73–3.49 mSv) the tin-filtered LD-CT showed a significantly lower effective dose than the standard CT with median 8.02 mSv (6.42–9.27 mSv) (*p* < 0.001), which is equivalent to a dose reduction of 59.9%. The values for CTDIvol, DLP and other scan parameters for both the standard CT and the tin-filtered LD-CT are listed in Table 1.

**Table 1:** Acquisition parameters of patient CT scans

	Standard CT	Low-dose Sn CT	*P* Value*
Tube current (kV)	120	Sn 140	NA
Tube current–time product (mAs)	123–437	243–392	NA
CTDIvol (mGy)	18.24 (15.42–21.48)	7.25 (6.78–7.66)	< .001
DLP (mGy* cm)	445.6 (356.45–515.3)	179.1 (152.08–193.83)	< .001
Effective dose (mSv) †	8.02 (6.42–9.27)	3.22 (2.73–3.49)	< .001
Scan length (mm)	243 (211–217.5)	242.5 (210.5–273.75)	.07

^†^ Estimation of effective dose (mSv): DLP multiplied with a standard conversion factor k for the adult lumbar spine (k = 0.018 mSv/mGy*cm)

Values are displayed as median with 25^th^ percentile and 75^th^ percentile in parentheses

^*^ Calculation of *P* values with Wilcoxon signed-rank test

Abbreviations: CTDIvol = volume CT dose index, DLP = dose length product, kV = kilo volt, mAs = milliampere seconds, mGy = milligray, mSv = millisievert, NA = not applicable, Sn = tin filter

### Qualitative image analysis

**Fig. 2:**
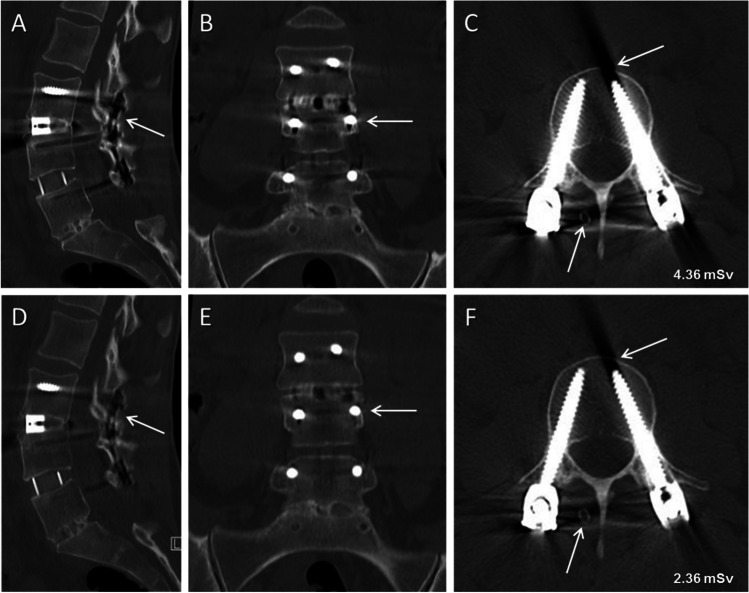
A 38-year-old female with lumbar instrumentation L3-5. Reformatted sagittal (**A** and **D**), coronal (**B** and **E**) and axial (**C** and **F**) CT images of the 120 kV standard CT (**A**-**C**) and of the 140 kV low-dose CT with tin filtration (**D**-**F**). Both datasets show clear depiction of anatomy and weak image noise. The tin-filtered low-dose CT demonstrates significantly less image artifacts (arrows) compared to the standard CT

For standard CT and tin-filtered low-dose CT, both readers rated the depiction of anatomy as good: with median 4 (Q1–Q3: 4–4) for standard CT and median 4 (3–4) for tin-filtered LD-CT, reader 1 reported a difference in quality (*p* < 0.001), while reader 2 observed no difference with median 4 (3–4) and 4 (3–4) (*p* = 0.84), respectively. Reader 1 and 2 rated image noise lower for standard CT compared to tin-filtered LD-CT: median 4 (4–4) vs. 3 (3–4) (*p* < 0.001) and 4 (3–4) vs. 3 (3–4) (*p* = 0.01), respectively. A highly significant difference between datasets was overserved by both readers regarding image artifacts: Reader 1 described strong artifacts for standard CT (median 2 (2–3)) and moderate artifacts for tin-filtered low-dose CT (median 3 (3–4)) (*p* < 0.001). Reader 2 also observed less image artifacts for tin-filtered LD-CT, with median 4 (4–4) compared to standard CT with median 3 (2–4) (*p* < 0.001) (Fig. 2).

### Quantitative analysis and CT artifacts

Regarding trabecular bone, CT values were measured for both datasets in 66 of 74 participants, in the other eight participants the L3 was artifact-impaired. With a median of 122 HU (80.5–150.3 HU) the values of trabecular bone were higher for standard CT than for the tin-filtered LD-CT with median 94 HU (62–121.5 HU) (*p* < 0.001). No difference was observed between datasets for psoas muscle (*n* = 74) with median 52 HU (48–54.3 HU) and 51 HU (48–54.3 HU) (*p* = 0.7), respectively. Image noise (*n* = 74) was significantly lower for standard CT with median 32 HU (28–35 HU) compared to tin-filtered low-dose CT with median 41.5 HU (37–45 HU) (*p* < 0.001). SNR with median 3.55 (2.59–4.84) and CNR with median 1.03 (0.51–1.66) were higher for standard CT in comparison to the tin-filtered low-dose CT with median SNR 2 (1.38–3.11) and median CNR 0.6 (0.22–0.93) (*p* < 0.001 and *p* < 0.001). Between standard CT and tin-filtered LD-CT there was no difference in dose efficiency (FOM of median 0.12 (0.04–0.54) vs. median 0.12 (0.02–0.39) (*p* = 0.11)).

**Fig. 3:**
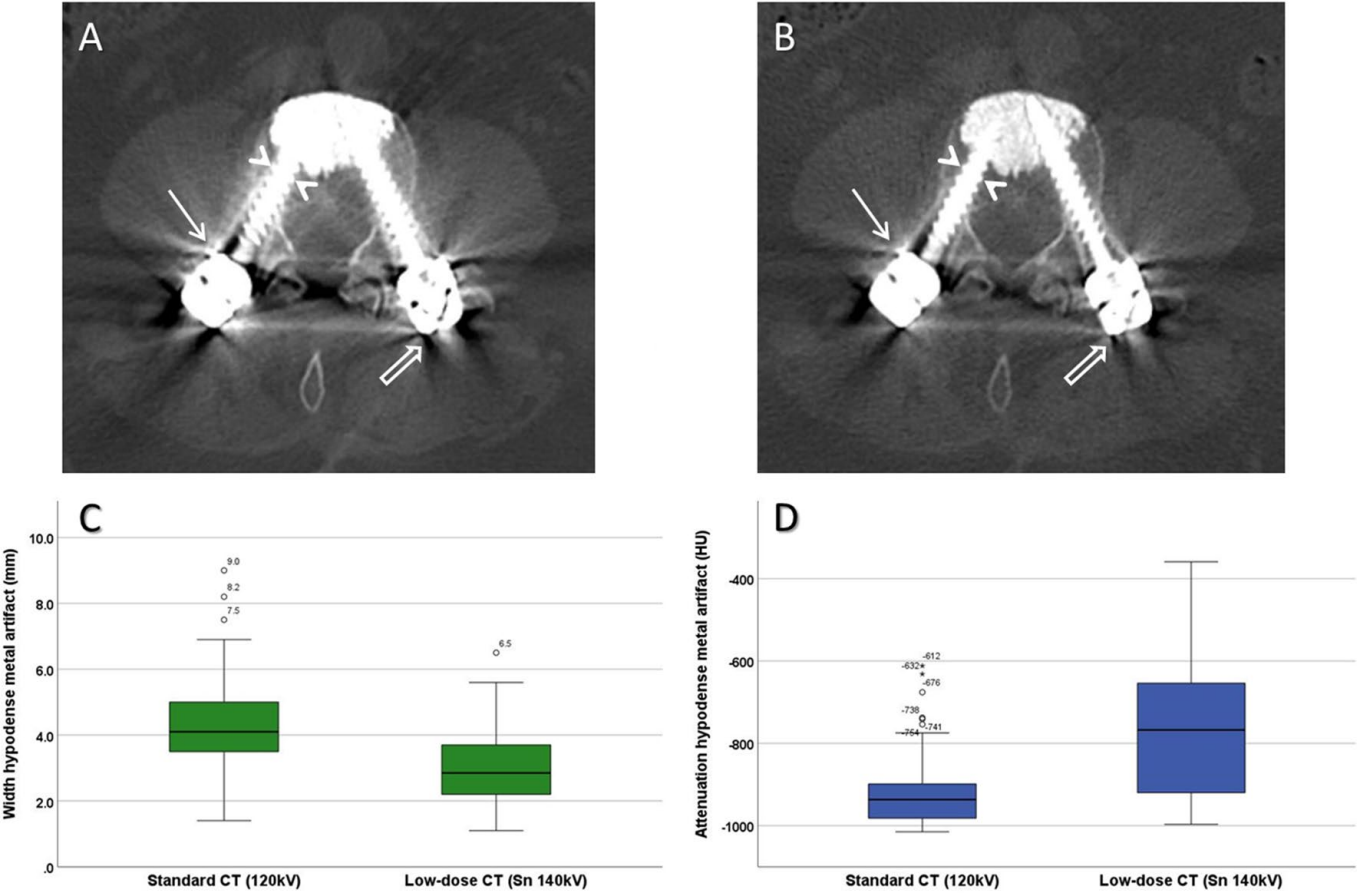
A 74-year-old female with cement-augmented lumbar instrumentation. Reformatted axial CT image of the standard CT with 120 kV (**A**) and of the low-dose CT with tin filtration (Sn 140 kV; **B**) both show bilateral pedicle screws and cement in the anterior vertebral body at level L4. Hyperdense (arrow) and hypodense (open arrow) streak artifacts are significantly reduced for the tin-filtered low-dose CT compared to the standard CT and depiction of the screw-bone interface is much clearer in **B** (arrowheads). Visualization of the spinal canal, the facet joints, and the dorsal paraspinal and psoas muscle is less artifact-impaired in **B** than in **A**. Reader 1 and 2 rated image artifacts higher for standard CT than for the tin-filtered low-dose CT (very strong vs. moderate and strong vs. weak, respectively). Quantitative comparison of the width (**C**) and the attenuation (**D**) of the most prominent hypodense artifact around the metallic spine implant between standard CT and tin-filtered low-dose CT of the study population

However, for the tin-filtered LD-CT the width of the most prominent hypodense artifact around the metallic spine implant was significantly smaller with median 2.9 mm (2.2–3.7 mm) compared to the standard CT with median 4.1 mm (3.5–5 mm) (*p* < 0.001). Regarding the CT value of the most prominent hypodense artifact around the metallic spine implant, significantly higher CT numbers were observed for the tin-filtered LD-CT (median -767.5 HU (-920 HU – -650.5 HU) than for the standard CT (median -937 HU (-982.3 HU – -899 HU) (*p* < 0.001) (Fig. 3).

### Imaging findings

**Fig. 4:**
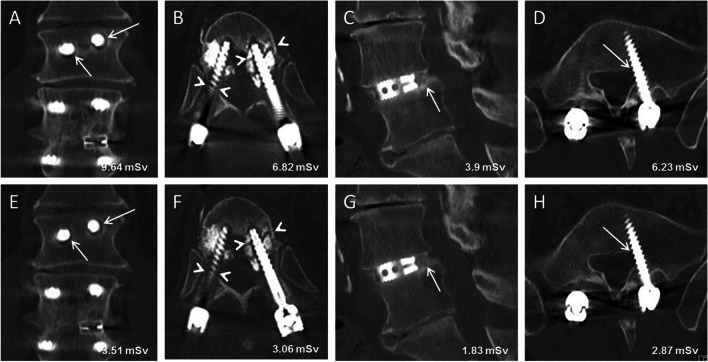
Standard 120 kV CT (**A**-**D**) and corresponding 140 kV tin-filtered low-dose CT (**E**–**H**) in 4 different patients after lumbar instrumentation. 60-year-old male (**A** and **E**) with bilateral loosening of pedicle screws at level L1 (arrows). 68-year-old male (**B** and **F**) with bilateral pedicle fracture at level Th12 (arrowheads). 80-year-old male (**C** and **G**) with osseous fusion posterior to the cage at level L4/5 (arrows). 27-year-old male (**D** and **H**) with left pedicle screw traversing lateral recess at level S1 (arrows)

**Fig. 5:**
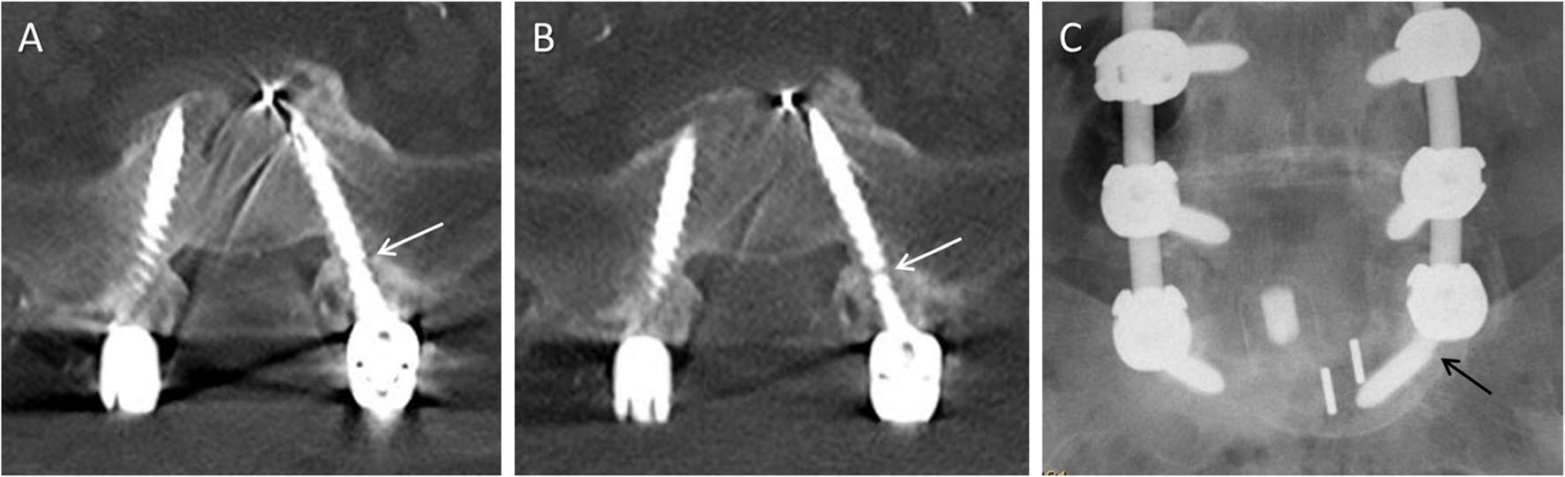
A 78-year-old female with lumbar instrumentation L3-S1. On the reformatted axial CT image of the tin-filtered low-dose CT (**B**) a fracture of the left S1 pedicle screw (arrow) is visible, which was missed by both readers on the standard CT (**A**) because of masking due to stronger artifacts. The fracture of the left S1 pedicle screw (arrow) is also visible on the anteroposterior radiograph of the lumbar spine (**C**)

Table 2 shows the prevalence of assessed imaging findings for both the standard CT and the tin-filtered LD-CT. Results of reader 1 and 2 are shown separately. There was no statistically significant difference in the detection rate of each imaging finding between standard CT and tin-filtered low-dose CT for reader 1 (*p*: 0.63 – > 0.99) and reader 2 (*p*: 0.23 – > 0.99) (Fig. 4). Of note, one fracture of a pedicle screw was observed by reader 1 and 2 on the tin-filtered LD-CT, and was missed by both readers on the standard CT. The screw fracture was verified on additional lumbar radiographs, which were acquired 3 weeks before the CT scans (Fig. 5). Interreader agreement between the musculoskeletal radiologist and the orthopedic spine surgeon was substantial for both standard CT (ĸ = 0.67) and tin-filtered low-dose CT (ĸ = 0.65).

**Table 2:** Imaging findings of patient CT scans

	Standard CT Reader 1	Low-dose Sn CT Reader 1	*P* Value* Reader 1	Standard CT Reader 2	Low-dose Sn CT Reader 2	*P* Value* Reader 2
Pedicle screw associated fracture	4	4	> .99	2	1	> .99
Fracture at other location	1	1	> .99	1	0	> .99
Break of pedicle screw	0	1	> .99	0	1	> .99
Osteolytic bone resorption around pedicle screw	24	26	.63	34	37	.7
Loosening of pedicle screw	12	13	> .99	15	16	> .99
Segmental osseous fusion	59	59	> .99	49	54	.23
Pedicle screw traversing lateral recess	7	6	> .99	12	9	.45

* Calculation of P values with McNemar test

Abbreviations: Sn = tin filter

Regarding the different image findings, for *pedicle screw associated fracture* a substantial agreement was observed for standard CT (ĸ = 0.65) and only a fair agreement for tin-filtered low-dose CT (ĸ = 0.38). The standard CT and the tin-filtered LD-CT showed similar agreement for *osteolytic bone resorption around pedicle screw* (ĸ = 0.38 (fair) and (ĸ = 0.37 (fair)) and *loosening of pedicle screw* (ĸ = 0.86 (excellent) and (ĸ = 0.87 (excellent)). For the tin-filtered LD-CT agreement was substantial for both *segmental osseous fusion* (ĸ = 0.66) and *pedicle screw traversing lateral recess* (ĸ = 0.63), while for the standard CT effect size was moderate each (ĸ = 0.59 and ĸ = 0.58). Interreader agreement was excellent for *fracture at other location* for standard CT (ĸ = 1) and for *fracture of pedicle screw* for tin-filtered low-dose CT (ĸ = 1).

## Discussion

Tin-filtered low-dose CT of the lumbar spine with metal implants was performed with 60% dose saving compared to standard clinical CT (median effective dose 3.22 mSv vs. 8.02 mSv) and showed superior metal artifact reduction. Despite an increase in noise for the tin-filtered LD-CT, the detection of relevant image findings was not different to the standard clinical CT and agreement between readers was similar (ĸ = 0.65 vs. ĸ = 0.67).

Several studies showed effective dose reduction for different body regions with the use of tin prefiltration CT [9, 11]. For the chest, Messerli et al. reduced the radiation dose to the level of chest radiographs by implementing the tin filter in their CT protocol (mean effective dose 0.13 mSv). Computer-aided detection of solid pulmonary nodules was not impaired in comparison to the standard CT protocol (mean effective dose 1.8 mSv) [9].

For the abdomen, Leyendecker et al. reduced the effective dose of contrast-enhanced 100 kV abdominopelvic CT to mean 1.14 mSv with the use of a tin-filtered CT protocol, compared to 5.99 mSv for the standard clinical CT. No significant difference in performance was observed for abdominal findings between protocols [11]. The results of our study were in accordance: in patients with metal implants of the lumbar spine the tin-filtered low-dose CT performed similar to the clinical standard CT in detecting pathology and surgery related complications (*p*: 0.23 – > 0.99).

In a previous study, Stern et al. showed the feasibility of tin-filtered LD-CT of the osseous pelvis at a dose equivalent to standard radiographs (median effective dose 0.38 mSv). Compared to the clinical standard CT with a median effective dose of 2.31 mSv, the low-dose protocol with tin prefiltration demonstrated clear depiction of anatomy and accurate detection of osseous pathologies. However, image noise was higher for the tin-filtered low-dose CT, a finding which we also observed for the tin-filtered LD-CT of the lumbar spine with metal implants (median noise 41.5 HU vs. 32 HU; *p* < 0.001) [10.1007/s00330-021-07824-x].

Furthermore, Stern et al. also discovered less image artifacts (scattering and beam hardening) around dense structures such as cortical bone for the tin-filtered LD-CT compared to the standard CT [10.1007/s00330-021-07824-x]. The results of the current study were in accordance, as in comparison to the standard CT, the tin-filtered LD-CT of the instrumented lumbar spine showed significantly less artifacts (smaller width (*p* < 0.001) and higher CT number (*p* < 0.001) of the most prominent hypodense artifact around the metallic spine implant). Zhou et al. reported artifact reduction with tin-filtered scans on a photon-counting-detector CT system with limited availability whereas we used a widely available energy-integrating-detector CT: for photon-counting-detector CT the difference in the mean CT number between hypodense artifact and non-artifact regions was smaller (440 HU) and mean artifact size decreased (0.45 mm) compared to the energy-integrating-detector CT (539 HU and 1.11 mm; *p* < 0.01 and *p* < 0.001, respectively). However, the sample size was small, with only 9 patients with metal implants of the lumbar spine [14]. In a pelvic cadaver study evaluating different techniques for their effectiveness in metal artifact reduction on energy-integrating-detector CT, Hackenbroch et al. showed the best overall performance for the tin-filtered 150 kV CT in combination with software-based iterative metal artifact reduction (iMAR Sn 150 kV) [13]. However, they were confronted with new artifacts generated by iMAR, which impaired image quality. For that reason, we decided not to use additional iMAR in our study.

Huflage et al. investigated different metal artifact reduction techniques in low dose imaging on an energy-integrating-detector CT using 2 cadavers with different metal implants (none had metallic implants of the lumbar spine). They found that the tin prefiltration CT with 150 kV (Sn 150 kV) performed best for reduction of hyperdense streak artifacts and equally good as the virtual monoenergetic imaging at 150 keV for reduction of hypodense streak artifacts, but with beneficial delineation of cortical boundaries [12]. The results of our in vivo study with metallic lumbar spine implants were in accordance with reduced attenuation in areas of the hypodense streak artifacts for images of the tin-filtered LD-CT compared to the standard CT (-767.5 HU vs. -937 HU).

Through the implementation of the tin-filtered LD-CT of the instrumented lumbar spine with an achieved dose reduction of 60% (median effective dose 3.22 mSv) we approached the reported dose levels of lumbar spine radiographs. Simpson et al. assessed the total effective dose of radiographs of the lumbar spine with 3.7 mSv, consisting of 2.2 mSv for the anteroposterior and 1.5 mSv for the lateral radiograph [5]. With the implementation of tin-filtered low-dose CT we are convinced to reduce the dose gap between CT and radiographs of the lumbar spine with the benefit of detailed cross-sectional anatomic information, which helps to establish the correct diagnosis in equivocal radiographic cases.

A limitation of our study was that we did not assess other techniques for metal artifact reduction besides tin prefiltration. Since our study included patients, who already received two CT scans (the clinical standard CT and the tin-filtered LD-CT scan for the study), further CT scans with a different technique (e.g., dual energy CT) would not have been justifiable, because of the potential risk of radiation induced damage. Furthermore, since the tin-filtered LD-CT comprises higher perceivable noise, there might be an impact on the blinded reading process and a potential bias for the readers.

In summary, with tin prefiltration and a low-dose protocol, 60% dose saving was achieved for CT of the lumbar spine with metal implants without compromise in detection of pathology and surgery related complications. Furthermore, reduction of metal artifacts was superior for the tin-filtered low-dose CT compared to the clinical standard protocol without tin prefiltration.

### Supplementary Information

Below is the link to the electronic supplementary material.

Supplementary file1 (DOCX 72 KB)

## Abbreviations

CNR: Contrast-to-noise ratio
CTDIvol: Volume computed tomography dose index
DLP: Dose length product
FOM: Figure of merit
HU: Hounsfield unit
kV: Kilo volt
LD-CT: Low-dose computed tomography
mAs: Milliampere seconds
mGy: Milligray
mSv: Millisievert
ROI: Region of interest
Sn: Tin filter
SNR: Signal-to-noise ratio
