# Supplementary figures and images for: Superior metal artifact reduction of tin-filtered low-dose CT in imaging of lumbar spinal instrumentation compared to conventional computed tomography
Source: Skeletal Radiol. 2023 Oct 7;53(4):665–73. doi: 10.1007/s00256-023-04467-5 (PMC10858831; doi:10.1007/s00256-023-04467-5)

Supplementary information

**Definition of ratings on 4-point Likert scale**
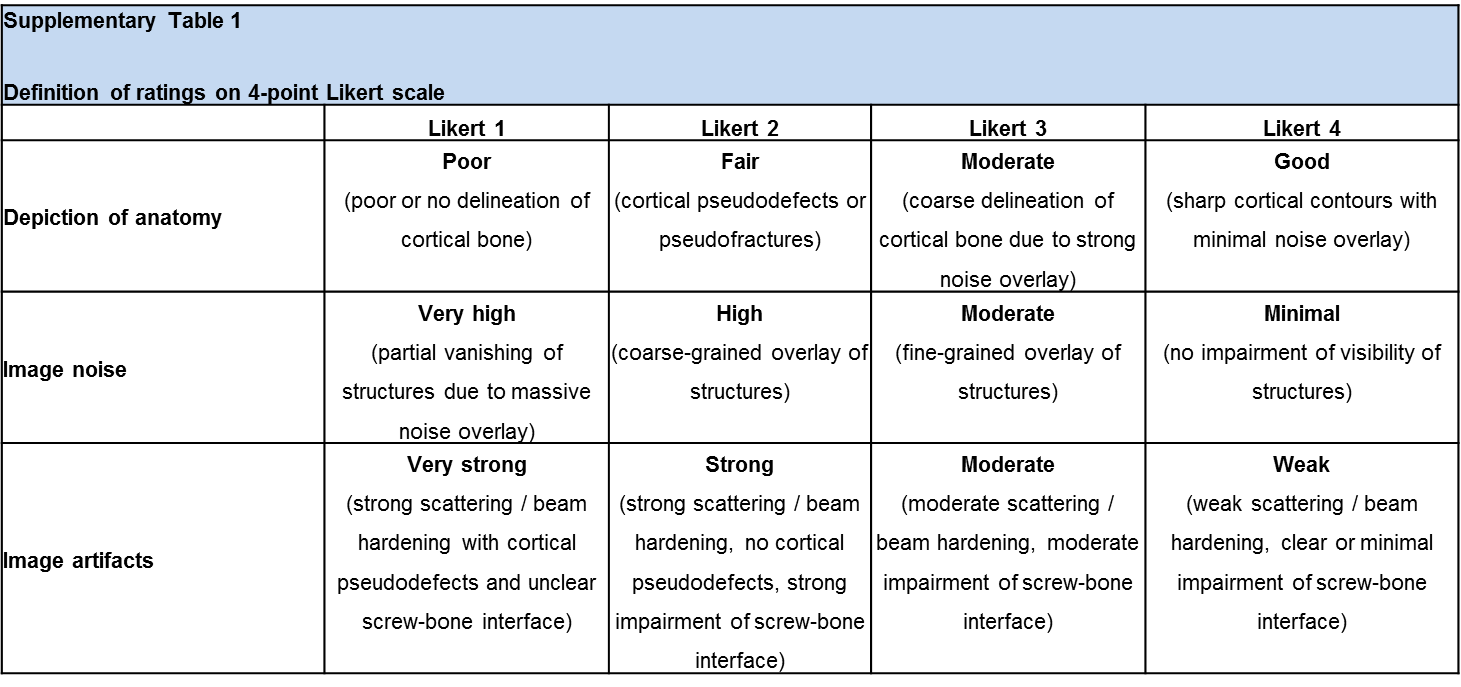

Supplement: Supplementary file 1 — Supplementary file1 (DOCX 72 KB) [file 256_2023_4467_MOESM1_ESM.docx]
